# Vaccination with inhibin-α provides effective immunotherapy against testicular stromal cell tumors

**DOI:** 10.1186/s40425-017-0237-2

**Published:** 2017-04-18

**Authors:** Robert Aguilar, Justin M. Johnson, Patrick Barrett, Vincent K. Tuohy

**Affiliations:** 1 Department of Immunology, NB30, Lerner Research Institute, Cleveland Clinic, 9500 Euclid Avenue, Cleveland, OH 44195 USA; 2 Department of Biology, Cleveland State University, Cleveland, OH USA; 3 Western Reserve Academy, Hudson, OH USA; 4 Department of Molecular Medicine, Cleveland Clinic Lerner College of Medicine of Case Western Reserve University, Cleveland, OH USA

**Keywords:** Testicular cancer, Stromal cell cancer, Cancer vaccines, Leydig cell tumors, Sertoli cell tumors

## Abstract

**Background:**

Testicular cancer is the most common male neoplasm occurring in men between the ages of 20 and 34. Although germ-line testicular tumors respond favorably to current standard of care, testicular stromal cell (TSC) tumors derived from Sertoli cells or Leydig cells often fail to respond to chemotherapy or radiation therapy and have a 5-year overall survival significantly lower than the more common and more treatable germ line testicular tumors.

**Methods:**

To improve outcomes for TSC cancer, we have developed a therapeutic vaccine targeting inhibin-α, a protein produced by normal Sertoli and Leydig cells of the testes and expressed in the majority of TSC tumors.

**Results:**

We found that vaccination against recombinant mouse inhibin-α provides protection and therapy against transplantable I-10 mouse TSC tumors in male BALB/c mice. Similarly, we found that vaccination with the immunodominant p215-234 peptide of inhibin-α (Inα 215-234) inhibits the growth of autochthonous TSC tumors occurring in male SJL.AMH-SV40Tag transgenic mice. The tumor immunity and enhanced overall survival induced by inhibin-α vaccination may be passively transferred into naive male BALB/c recipients with either CD4+ T cells, B220+ B cells, or sera from inhibin-α primed mice.

**Conclusions:**

Considering the lack of any alternative effective treatment for chemo- and radiation-resistant TSC tumors, our results provide for the first time a rational basis for immune-mediated control of these aggressive and lethal variants of testicular cancer.

## Background

Testicular cancers are the most common solid tumors occurring in young men aged 20–34 with a median age at diagnosis of 33 [1, 2]. The vast majority of testicular cancers are germ cell tumors that have a 5-year overall survival rate exceeding 95% when treated early with surgery, chemotherapy, and/or radiation therapy [1, 2]. However, a small percentage of testicular tumors develop in the hormone-producing cells of the stroma including Leydig and Sertoli cell tumors that respond poorly to current standard of care and have 5-year survival rates of 91% and 77%, respectively [3]. Thus, there is a great need for more effective treatments against testicular stromal cell (TSC) tumors. To this end, we have developed an immunotherapeutic approach for providing improved control over TSC tumors and enhanced overall survival.

Our approach involved immune targeting of inhibin-α, a gonadal protein that belongs to the transforming growth factor beta superfamily and plays a role in regulating secretion of pituitary follicle stimulating hormone through a negative feedback mechanism [4]. Inhibin-α is a 366 amino acid protein with a molecular mass of 39.56 kDa (GenBank: EDL00422.1). Inhibin-α restricts the production of mature ovarian follicles in mammalian females and regulates spermatogenesis, steroidogenesis, and germ cell development in males [4]. Inhibin-α is produced by normal TSC and is expressed and produced in the majority of human and canine TSC tumors [5–7]. Moreover, several studies indicate that inhibin-α is a useful marker for human TSC tumors with intense immunohistochemical staining of inhibin-α occurring typically in >90% of Leydig and Sertoli cell tumors but in only about 10% of testicular germ cell tumors [5, 8, 9]. Therefore, inhibin-α stands out prominently as a potentially useful vaccine target for providing immunotherapy against TSC tumors.

Here we show that vaccination of male BALB/c mice with recombinant mouse inhibin-α (rmInα) induces a type-1/type-17 proinflammatory T cell response sufficient to inhibit the growth of transplantable I-10 TSC tumor cells using both prevention and treatment protocols. Tumors from mice vaccinated with rmInα are extensively infiltrated with CD3+ T cells, many of which are activated CD4 + CD44+ T cells. The immunity against TSC tumors could be transferred into naive recipient BALB/c males using inhibin-α primed CD4+ T cells, B220+ B cells, or sera but not with inhibin-α primed CD8+ T cells. We also found that vaccination with the p215-234 immunodominant peptide of mouse inhibin-α (Inα 215-234) provides significant inhibition of autochthonous TSC tumor growth occurring spontaneously in SJL.AMH-SV40Tag transgenic mice. Taken together, our data support the view that vaccination against inhibin-α has the potential to provide significant immunotherapy against TSC tumors that may prove useful in the adjuvant setting for control of this aggressive form of testicular cancer and for enhancing the overall survival of patients with these tumors.

## Methods

### Generation of recombinant mouse inhibin-α (rmInα)

Total RNA was purified from testes of 8 week old BALB/c male mice using the RNeasy Mini Kit (Qiagen, Valencia, CA), and the RNA was stabilized in RNAlater (Qiagen). cDNA was generated with random hexamers using the SuperScript III First-Strand Synthesis SuperMix (ThermoFisher Scientific, Waltham, MA). Inhibin-α cDNA was amplified using the AmpliTaq Gold DNA Polymerase LD kit (ThermoFisher Scientific) with CCTAGGCAGGAAGAGCACAG as forward primer and ACCTCCATCTGAGGTGGTTC as reverse primer. The inhibin-α cDNA sequence was inserted into the NdeI-Bam HI sites of the pET-3a expression vector (GeneArt AG, Regensburg, Germany) thereby providing a C-terminal 6 × His-tagged recombinant protein after plasmids containing this insert was transformed in BL21 Star *E. coli* (Lucigen, Middleton, WI). High level expression colonies were selected following induction with isopropyl β-D-1-thiogalactopyranoside (IPTG; Amresco, Solon, OH) and were sequenced for confirming proper orientation and alignment. The 6 × His-tagged protein was purified under denaturing conditions using nickel-nitrilo triacetic acid (Ni-NTA) affinity chromatography (Qiagen). The purity of affinity purified rmInα was gauged by SDS-PAGE and Western blot analysis using mouse inhibin-α antibody at 1/200 dilution and secondary detection antibody at 1/5,000 dilution (Santa Cruz Biotechnology, Dallas, TX). Prior to use in vitro, the inhibin-α protein was further purified by reverse phase high performance liquid chromatography (HPLC) to yield endotoxin-free protein [10]. Levels of endotoxin were < 5 pg/mg recombinant protein.

### Generation of SJL.AMH-SV40Tag transgenic mice and autochthonous TSC tumors

The AT-t94 transgenic mouse was generously provided by Dr. Jean-Yves Picard, Biologie Fonctionnelle et Adaptative Université, Paris, France. This mouse expresses a fusion construct containing 3.6 kb of the 5' flanking region of the human anti-Müllerian hormone (AMH) gene upstream of the SV40 proto-oncogene encoding the large transforming antigen (SV40Tag) [11, 12]. Female AT-t94 mice develop a high incidence of autochthonous granulosa cell tumors and male AT-t94 transgenic mice develop a high incidence of autochthonous TSC tumors [11, 12]. Male AT-t 94 transgenic mice expressing the H-2^b^ haplotype of the major histocompatibility complex (MHC) were mated at the Cleveland Clinic with female SJL/J (H-2^s^) mice obtained commercially (Jackson Laboratory, Bar Harbor, ME). The resultant SJL × AT-t94 (H-2^b,s^) transgenic offspring were backcrossed for over 20 generations to SJL/J mice producing SJL.AMH-SV40Tag (H-2^s^) transgenic mouse used in the current study. Female SJL.AMH-SV40Tag transgenic mice develop granulosa cell tumors starting at 8-10 months of age and show an incidence of affected ovaries that exceeds 90% by 18 months of age [13]. Moreover, the emergence and growth of autochthonous granulosa cell tumors in female SJL.AMH-SV40Tag transgenic mice is inhibited by vaccination with the IA^s^-restricted Inα 215-234 peptide of mouse inhibin-α [13, 14]. In our hands, male SJL.AMH-SV40Tag transgenic mice develop unilateral and bilateral Leydig cell tumors at around 75 weeks of age and are able to respond to the IA^s^-restricted Inα 215-234. SJL.AMH-SV40Tag transgenic mice were identified by RT-PCR amplification of the human AMH promoter from tail DNA.

### The I-10 mouse testicular cancer cell line and the transplantable TSC tumor model

The I-10 (ATCC® CCL83™) mouse testicular cancer cell line was purchased from the American Type Culture Collection (ATCC, Manassas, VA). I-10 cells are hyperdiploid, epithelial-like Leydig tumor cells derived from male BALB/c mice using a single-cell plating technique [15, 16]. I-10 cells were grown in F-12K media (ATTC #30-2004) supplemented with 2.5% heat inactivated fetal bovine serum and 15% heat inactivated horse serum (ThermoFisher Scientific). Prior to use, all media were filtered through a 0.2 μm Nalgene Rapid-Flow Disposable Bottle Top Filter (ThermoFisher Scientific). The I-10 cells were culture as a single-cell suspension in 75-cm^2^ tissue culture flask (ThermoFisher Scientific) and cultured at 37 °C in humidified air and 5% CO_2_ with intermittent feeding using warm fresh media. At 70–75% confluence, adherent cells were disrupted mechanically and enzymatically by adding 10 ml F-12K media containing 0.25% trypsin and 0.02% EDTA (ThermoFisher Scientific). After centrifugation and thorough washing in PBS, pelleted cells were recultured in supplemented F-12K media or resuspended in PBS for subcutaneous inoculation of 2 × 10^4^ cells in the lumbar region of 8–10 week-old BALB/cJ males purchased commercially (Jackson Laboratory). Tumors were measured daily by Vernier caliper and mice were euthanized when tumors reached 17 mm in either length or width. BALB/c mice typically developed palpable tumors within 28 days after inoculation.

### Peptide synthesis

Inα 215-234, FLVAHTRARAPSAGERARRS, was synthesized by the Molecular Biotechnology Core Facility of the Lerner Research Institute using standard solid phase methodology and FMOC side chain-protected amino acids. The peptide was purified > 97% by reverse phase HPLC, and amino acid composition was confirmed by mass spectrometry.

### Mice and immunization

Male BALB/cJ mice were obtained commercially (Jackson Laboratory) at 6-7 weeks of age and usually immunized at 8–10 weeks of age by subcutaneous injection in the abdominal flank with 100 μg of rmInα protein in 200 μl of an emulsion of equal volumes of water and complete Freund's adjuvant (CFA) containing 400 μg of *Mycobacteria tuberculosis* H37RA (Difco, Detroit, MI). In the treatment protocol, all experimental BALB/cJ mice were inoculated on the same day with 2 × 10^4^ I-10 cancer cells thereby ensuring an equal initial tumor load. When the first tumor became palpable in any mouse, all mice in that treatment group were vaccinated. Although not all mice had palpable tumors on the day of vaccination, all had palpable tumors within a day or two thereafter. Male SJL.AMH-SV40Tag transgenic mice were immunized at 8–10 weeks as described above but with 100 μg of Inα 215-234 peptide. All mice were euthanized by asphyxiation with CO_2_ followed by cervical dislocation.

### Proliferation assays

To characterize the immune response to rmInα and Inα 215-234, lymph node cells (LNC) were removed 10 days after immunization or spleens were removed 8 weeks after immunization. Each population was cultured with immunogen at various doses in triplicate 96-well flat-bottom microtiter Falcon plates (BD Biosciences, San Jose, CA) at 3 × 10^5^ cells/well in a total volume of 200 μl of DMEM (Mediatech, Manassas, VA) supplemented with1% penicillin/streptomycin, 2% L-glutamine, 5% HEPES buffer (Invitrogen Life Technologies, Grand Island, NY), and 10% fetal bovine serum (Hyclone, Logan, UT). Positive control wells contained 2 μg/ml anti-mouse CD3 (BD Biosciences), negative control wells contained no antigen, and specificity controls contained grade VII ovalbumin (Sigma-Aldrich, St. Louis, MO) at various doses. In all cases, proliferation was determined after 96 h of culture when wells were pulsed with 1.0 μCi/well thymidine, [methyl-3H]-, specific activity: 6.7 Ci/mmol (PerkinElmer, Waltham, MA) and harvested 16 h later by aspiration onto glass fiber filters. Levels of incorporated radioactivity were determined by scintillation spectrometry. Results are expressed as mean counts per minute (cpm) of experimental cultures with antigen divided by mean cpm of cultures without antigen (stimulation index). In all proliferation assays, mean cpm of cultures without antigen ranged between 500 and 2000 cpm.

### ELISPOT assays

Ten days after immunization with either rmInα or Inα 215-234, frequencies of type-1, type-2, and type-17 T cells responding to each immunogen were determined by ELISPOT analysis using capture/detection antibody pairs for interferon-gamma (IFNγ), IL-5, and IL-17 (ThermoFisher Scientific), respectively. Duplicate or triplicate wells containing 5 × 10^5^ LNC were cultured with 50 μg/ml immunogen or the irrelevant control antigen, grade VII ovalbumin (Sigma-Aldrich) in ELISPOT plates (Millipore, Billerica, MA) pre-coated with capture antibodies in 200 μl/well total culture volume in DMEM (Mediatech) supplemented as described above. At 72 h of culture, wells were treated with corresponding biotinylated detection antibodies and after overnight incubation and washing, spots were visualized by sequential treatment with alkaline phosphatase-conjugated streptavidin and 5-bromo-4-chloro-3-indolyl phosphate substrate (R&D Systems, Minneapolis, MN). The reaction was halted after 10 min by repeated washing with double-distilled deionized H_2_O, and spots were developed and counted using an ImmunoSpot S6 analyzer with proprietary ImmunoSpot 5.1 software (Cellular Technologies Limited, Shaker Heights, OH). In some experiments, CD4+ and CD8+ T cells were purified from primed LNC by negative selection using anti-CD4- and anti-CD8-coated magnetic beads and double passage through a MACS LS column using a MidiMACS cell separator (Miltenyi Biotec, San Diego, CA). The enriched T cells were activated with various doses of immunogen in cultures containing 3 × 10^5^ T cells/microtiter well and 5 × 10^5^ γ-irradiated (25 Gy) syngeneic splenocyte feeders.

### ELISA assays

Cytokine concentrations were determined by ELISA measurement of 48-h supernatants of 10-day-primed LNC cultured in supplemented DMEM at 5 × 10^6^ cells/well in 24-well flat-bottom Falcon plates (BD Biosciences) in the presence of 20 μg/ml antigen in a final volume of 2.0 ml/well. Affinity purified capture/detection antibody pairs and recombinant cytokines (ThermoFisher Scientific) were used to measure supernatant concentrations of IFNγ, IL-5, and IL-17. Absorbance was measured at 405 nm using a model 550 ELISA microplate reader (Bio-Rad Laboratories, Hercules, CA). Standard values were plotted as absorbance vs. cytokine concentration, and sample cytokine concentrations were determined as values within the linear part of the standard curve established using known concentrations of each cytokine.

### Antibody isotyping

Isotype-specific serum antibody titers to rmInα were determined using the mouse MonoAB ID/SP ELISA kit (Zymed Laboratories, South San Francisco, CA).

### Passive transfer of tumor immunity

Three weeks after immunization of BALB/c male mice with rmInα, CD4+ T cells, CD8+ T cells, and B220+ B cells, were enriched (>90%) from splenocytes by magnetic bead separation as described above. The enriched T cells were cultured in supplemented DMEM at 5 × 10^6^ cells/well in 24-well flat-bottom Falcon plates (BD Biosciences) in the presence of 20 μg/ml antigen in a final volume of 2.0 ml/well. Each well also contained 5 × 10^6^ γ-irradiated (25 Gy) syngeneic splenocyte feeders as antigen presenting cells. After 72 h of culture, cells were washed thoroughly and 2–3 × 10^7^ cells were injected intraperitoneally into naive recipient male BALB/c mice in a total volume of 200 μl PBS. For serum transfer experiments, recipients of rmInα-primed and ovalbumin-primed CD4+ T cells were bled at euthanasia by cardiac puncture. Cell-free sera were collected and 200 μl of pooled sera were injected intravenously into each naive BALB/c male recipient. After transfer of cells or serum, mice were inoculated later on the same day with I-10 TSC tumor cells as described above.

### Isolation of tumor infiltrating lymphocytes (TILs) and flow cytometry analysis

Leukocytes were isolated from I-10 tumors by digestion of minced tissue for 30 min at 37 °C in HBSS (ThermoFisher Scientific) containing 50 Kunitz Units (KU) of DNase I (Sigma-Aldrich) and 0.2 mg/ml collagenase II (ThermoFisher Scientific). Cells were then collected by discontinuous gradient centrifugation and the enriched TILs were triple-stained with commercially available (ThermoFisher Scientific) CD3-specific antibody conjugated with fluorescein isothiocyanate (FITC), CD44-specific antibody conjugated with cyanine 5 (Cy5), and either CD4-specific antibody conjugated with phycoerythrin (PE) or CD8-specific antibody conjugated with PE. Data were collected on 30,000 total events using a Becton-Dickinson FACSAria II flow cytometer (BD Biosciences) and analyzed using FlowJo software (FlowJo, Ashland, OR) after gating on the CD3+ population.

### Histology

Mouse testes and TSC tumors were fixed in 10% phosphate-buffered formalin (ThermoFisher Scientific) for 24 h and stored in 70% ethanol until processed for embedding in paraffin. Slides with multiple 6 μm tissue sections were stained with hematoxylin and eosin (Richard-Allan Scientific, Kalamazoo, MI), dehydrated in an ascending gradient of ethanol followed by xylene, and mounted in Cytoseal 60 (Stephens Scientific, Riverdale, NJ) for examination by light microscopy.

### Immunohistochemistry

Prior to immunostaining using the ImmunoCruz rabbit LSAB Staining System (Santa Cruz Biotechnology), tissue antigens were unmasked by heat treatment as per the manufacturer’s instructions. Briefly, prepared slides were treated with 10 mM sodium citrate buffer (pH 6) and heated to 95 °C. This process was then repeated with fresh buffer. After the slides cooled, they were washed with double distilled deionized H_2_O and the excess liquid was aspirated. After unmasking and blocking formalin-fixed 6 μm paraffin embedded tissues sections, antigens were detected using primary antibodies against luteinizing hormone receptor (LHR; Santa Cruz Biotechnology), AMH (Abcam, San Francisco, CA), SV40Tag (Santa Cruz Biotechnology), and mouse CD3 (Novacastra, Buffalo Grove, IL). All primary antibodies were used at a 1/1000 dilution.

### Biostatistical analysis

Differences between mean tumor weights and mean tumor areas were compared using the Student’s *t* test. Differences in Kaplan-Meier survival curves with 17 mm tumor endpoints were compared with the log-rank test.

## Results

### Generation of recombinant mouse inhibin-α (rmInα)

**Fig. 1:**
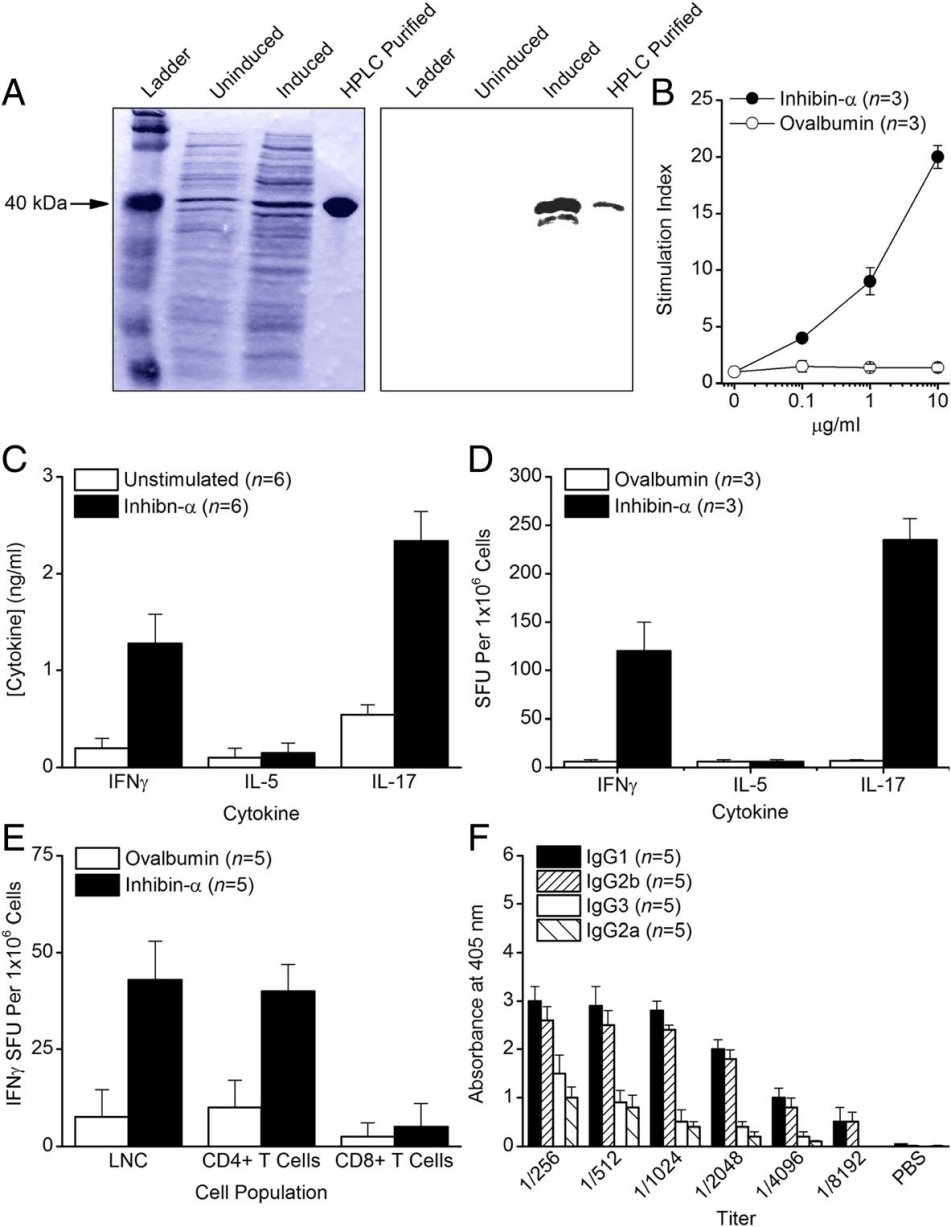
*Immunogenicity of recombinant mouse inhibin-α (rmInα).* **a** Coomassie blue staining (left panel) showed migration of affinity purified and HPLC purified rmInα on an SDS-PAGE gel at the anticipated molecular mass of 39.56 kDa. This correct molecular mass migration was confirmed by Western blot analysis of the HPLC purified protein using an antibody specific for mouse inhibin-α (right panel). **b** 8-10 week old male BALB/c mice were immunized with rmInα, and ten days later, LNC were tested for recall proliferative responsiveness. **c** Immunogenicity was confirmed by ELISPOT analysis of type-1, type-2, and type-17 T cells expressing IFNγ, IL-5, and IL-17, respectively. **d** Immunogenicity was also confirmed by ELISA measurement IFNγ, IL-5, and IL-17 concentrations in supernatants of rmInα primed LNC reactivated with antigen. **e** ELISPOT analysis of responses from purified T cell subpopulations showed that the response to rmInα was elicited predominantly from CD4+ T cells. **f** Serum antibody titers from BALB/c male mice 3 weeks after immunization with rmInα showed differential production of IgG isotypes. Error bars indicate ± SE

We selected inhibin-α as our vaccine target for immunotherapy against TSC tumors because it is expressed and produced in the majority of mouse and human TSC tumors [5–9]. Inhibin-α cDNA was inserted into the pET-3a expression vector to produce a 6 × His-tagged fusion protein after IPTG induction in BL21 Star *E. coli*. Coomassie blue staining showed that the induced protein and the HPLC purified protein migrate on SDS-PAGE at the anticipated molecular mass of 39.56 kDa (Fig. 1a, left panel). Western blotting using a primary antibody specific for mouse inhibin-α (Santa Cruz Biotechnology) confirmed the identity and purity of the generated rmInα protein (Fig. 1a, right panel).

### Immunogenicity of rmInα

To determine the immunogenicity of rmInα, 8–10 week old male BALB/c mice were immunized with rmInα, and ten days later, LNC were tested for recall proliferative responsiveness to the priming rmInα immunogen and to ovalbumin as a specificity control. The results showed that immunization with rmInα induced an antigen-specific response to the priming rmInα immunogen (Fig. 1b). This immunogenicity was confirmed by ELISPOT analysis that showed increased frequencies of proinflammatory type-1 and type-17 T cells producing IFNγ and IL-17, respectively, but no increased frequencies of T cells producing the regulatory cytokine IL-5 (Fig. 1c). In addition, ELISA analysis of supernatants from rmInα primed LNC reactivated with antigen showed production of the proinflammatory cytokines IFNγ and IL-17 but not production of the regulatory cytokine IL-5 (Fig. 1d). ELISPOT analysis of responses from purified T cell subpopulations showed that the response to rmInα was elicited predominantly from CD4+ T cells (Fig. 1e). Finally, high titer serum antibody responses to rmInα were evident by 3 weeks after immunization with IgG1 and IgG2b providing the predominant isotypes even at serum dilutions exceeding 1/8,000 (Fig. 1f).

### Vaccination against rmInα inhibits growth of transplantable TSC tumors

**Fig. 2:**
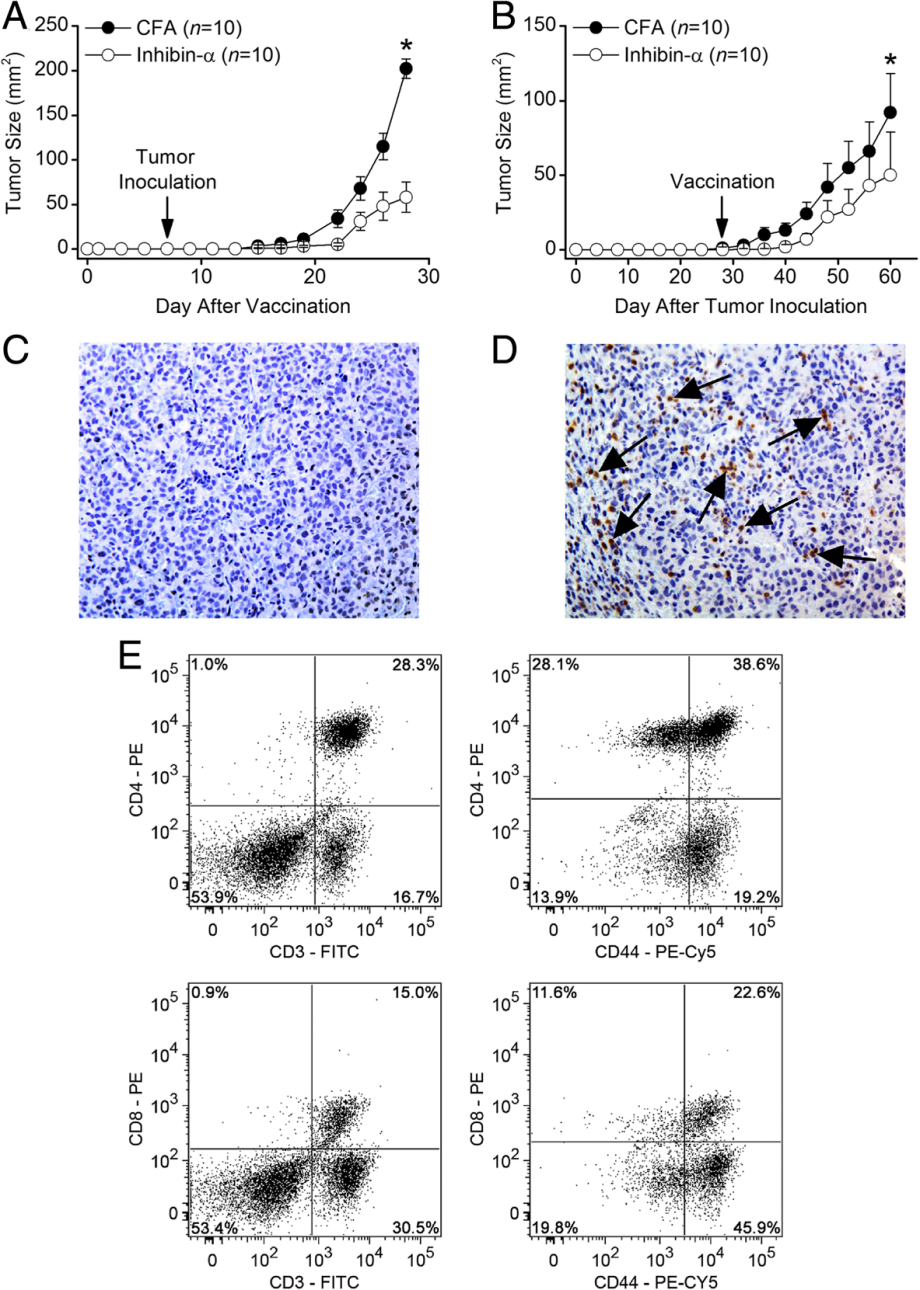
*Vaccination against rmInα inhibits growth of transplantable TSC tumors.* Significant inhibition of transplantable tumor growth occurred when rmInα vaccination occurred (**a**) prophylactically 8 days prior to inoculation with 1-10 TSC tumor cells or (**b**) as treatment of palpable I-10 TSC tumors. (**c**) Immunohistochemical analysis showed virtually no CD3+ T cells in I-10 TSC tumors 32 days after vaccination with CFA alone whereas (**d**) extensive CD3+ T cell infiltrates were evident in I-10 tumors taken from mice 32 days after vaccination against rmInα. Arrows point to examples of CD3+ T cells. (**e**) Flow cytometry analysis showed that 28.3% of the TILs were CD3 + CD4+ T cells (upper left panel) and 57.9% of these CD4+ T cells expressed the CD44 activation marker (upper right panel). Only 15% of TILs expressed the CD3 + CD8+ phenotype (lower left panel) and only 66.1% of these CD8+ T cells expressed the CD44 activation marker (lower right panel). Error bars indicate ± SE, and asterisks indicate significance

To determine whether rmInα vaccination could prevent the growth of transplantable TSC tumors, eight week-old male BALB/c mice were vaccinated with rmInα, and eight days later inoculated with 2 × 10^4^ I-10 TSC tumor cells. The results showed significant inhibition of tumor growth (*P* = 0.009) in rmInα vaccinated mice compared to control mice vaccinated with CFA alone (Fig. 2a). To determine whether rmInα vaccination could be used to treat established TSC tumors, tumor inoculated BALB/c mice were vaccinated when TSC tumors became palpable. Again, rmInα vaccination showed significant inhibition of tumor growth (*P* = 0.001) in rmInα vaccinated mice compared to control mice vaccinated with CFA alone (Fig. 2b). Immunohistochemical analysis showed virtually no CD3+ T cells in I-10 TSC tumors 32 days after vaccination with CFA alone (Fig. 2c) whereas extensive CD3+ T cell infiltrates occurred in I-10 TSC tumors 32 days after vaccination with rmInα (Fig. 2d). Thus, CD3+ tumor infiltrating lymphocytes (TILs) were consistently observed only in mice vaccinated with recombinant mouse inhibin-α, and these were the only mice that showed inhibited growth of TSC tumors. Flow cytometry analysis of the gated CD3+ population indicated that 28.3% of the TILs were CD3 + CD4+ T cells (Fig. 2e, upper left panel) and 38.6% of these CD4+ T cells expressed the CD44 activation marker (Fig. 2e, upper right panel). In contrast, only 15% of TILs were CD3 + CD8+ T cells (Fig. 2, lower left panel) and only 22.6% of these CD8+ T cells expressed the CD44 activation marker (Fig. 2e, lower right panel).

### Passive transfer of tumor immunity

**Fig. 3:**
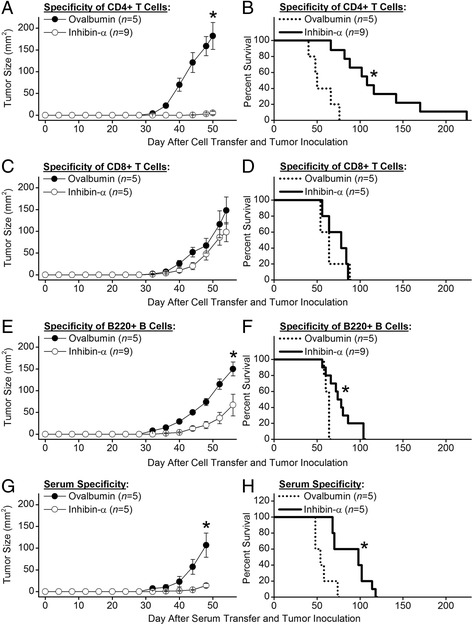
*Passive transfer of tumor immunity.* **a** Transfer of purified rmInα primed CD4+ T cells into naïve BALB/c recipient male mice provided highly significant protection against the growth of subsequently inoculated I-10 TSC tumors and (**b**) this protection resulted in a highly significant increase in overall survival compared to recipients of ovalbumin primed CD4+ T cells. **c** Transfer of rmInα primed CD8+ T cells failed to provide protection against the growth of I-10 tumors and (**d**) failed to enhance overall survival. (**e**) Transfer of rmInα primed B220+ B cells provided significant protection against the growth of I-10 TSC tumors and (**f**) significantly enhanced overall survival. **g** Transfer of serum from mice that received rmInα primed CD4+ T cells also provided significant protection against the growth of I-10 TSC tumors and (**h**) significantly enhanced overall survival. Error bars indicate ± SE, and asterisks indicate significance

To determine which lymphocytes were responsible for the induced tumor immunity, we transferred defined purified lymphocyte populations into naive tumor inoculated recipients. Eight week-old male BALB/c mice were immunized with either rmInα or with ovalbumin as a control immunogen. Three weeks later, primed CD4+ T cells, CD8+ T cells, and B220+ B cells were purified from splenocytes using magnetic bead separation. Prior to cell transfer, T cells were cultured for 72 h with the priming immunogen whereas B cells were transferred without prior restimulation. The purified cells were injected intraperitoneally into naive BALB/c male mice that were inoculated on the same day with I-10 TSC tumor cells. Our results showed that transfer of CD4+ T cells primed against rmInα provided highly significant protection (*P* < 0.001) against the growth of I-10 TSC tumors (Fig. 3a) and that this protection provided a highly significant increase (*P* < 0.001) in overall survival compared to recipients of ovalbumin primed CD4+ T cells (Fig. 3b). In contrast, transfer of rmInα primed CD8+ T cells failed to provide protection against the growth of I-10 TSC tumors (Fig. 3c) or enhancement of overall survival (Fig. 3d). In addition, we found that transfer of rmInα primed B220+ B cells provided significant protection (*P* = 0.003) against the growth of I-10 TSC tumors (Fig. 3e) and a significant increase (*P* = 0.001) in overall survival (Fig. 3f). Finally, transfer of serum from mice that received rmInα primed CD4+ T cells also provided significant protection (*P* = 0.001) against the growth of I-10 TSC tumors (Fig. 3g) and significantly enhanced (*P* = 0.001) overall survival (Fig. 3h). Thus, our data indicate that CD4+ T cells, B220+ B cells, and serum specific for rmInα are independently capable of significantly inhibiting growth of I-10 TSC tumors.

### Immunohistochemical analysis of autochthonous TSC tumors

**Fig. 4:**
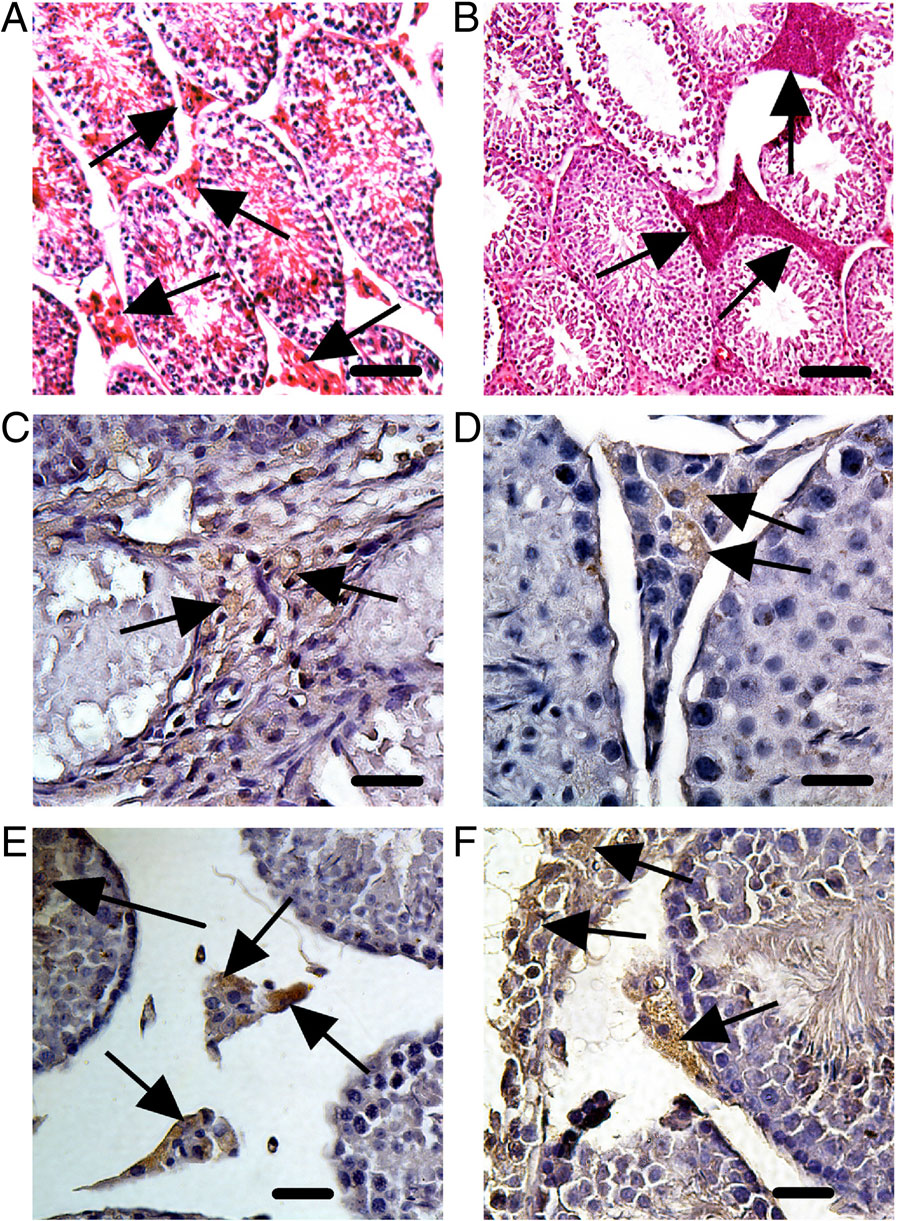
*Immunohistochemical analysis of autochthonous TSC tumors.* **a** Testis tissue taken from normal 75 week-old male SJL/J mice showed normal non-transformed Leydig cells in the interstitial space between the seminiferous tubules (arrows; bar = 25 μm). **b** Testis tissue taken from 75 week-old male SJL.AMH-SV40Tag mice showed transformed Leydig cell tumors in the interstitial space between the seminiferous tubules (arrows; bar = 25 μm), and positive staining for (**c**) luteinizing hormone receptor (LHR), a traditional Leydig cell marker (arrows; bar = 10 μm), **d** anti- Müllerian hormone (AMH), a traditional Sertoli cell marker (arrows; bar = 10 μm), **e** the transgene derived SV40Tag in Leydig cells (short arrows) and Sertoli cells (long arrow; bar = 25 μm), and (**f**) inhibin-α, a protein commonly expressed in both Leydig cell tumors and Sertoli cell tumors (arrows; bar = 25 μm)

Testis tissue taken from normal 75 week-old male SJL/J mice showed normal non-transformed Leydig cells in the interstitial space between seminiferous tubules (Fig. 4a). In contrast, testis tissue taken from 75 week-old male SJL.AMH-SV40Tag mice show transformed Leydig cell tumors in the interstitial space between seminiferous tubules (Fig. 4b). Immunohistochemical detection of the Leydig cell marker luteinizing hormone receptor (LHR) [17] showed localization in autochthonous TSC tumors from 75 week-old male SJL.AMH-SV40Tag transgenic mice (Fig. 4c). In addition, TSC tumors from 75 week-old male SJL.AMH-SV40Tag mice also expressed anti-Müllerian hormone (AMH; Fig. 4d), a protein traditionally associated with Sertoli cell tumors that would ordinarily develop within the seminiferous tubules and not in the interstitial space where Leydig cell tumors develop [18]. TSC tumors from male SJL.AMH-SV40Tag transgenic mice also expressed the SV40Tag from the transgene (Fig. 4e). Finally, the TSC tumors expressed inhibin-α (Fig. 4f), a protein expressed in the majority of Leydig cell tumors [5–9, 19] but also expressed in a substantial number of Sertoli cell tumors [5]. We estimate that about 88% of inhibin-α positive TSCs show malignant changes. Thus, the TSC autochthonous tumors that grow spontaneously in SJL.AMH-SV40Tag transgenic mice have many of the characteristics of Leydig cell tumors but also express AMH, a marker traditionally associated with tumors of Sertoli cell origin.

### Vaccination against rmInα inhibits growth of autochthonous TSC tumors

**Fig. 5:**
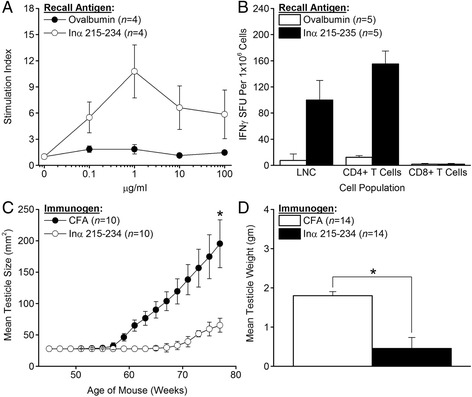
*Vaccination against rmInα inhibits growth of autochthonous TSC tumors.* Ten days after immunization of male SJL.AMH-SV40Tag (H-2^s^) transgenic mice with the immunodominant IA^s^-restricted Inα 215-234 peptide (**a**) LNC showed antigen-specific proliferation in response to various doses of Inα 215-234, and (**b**) whole LNC and purified CD4+ T cells but not CD8+ T cells showed high frequencies of antigen-specific IFNγ-secreting T cells in response to Inα 215-234. **c** Prophylactic Inα 215-234 vaccination of eight week-old male SJL.AMH-SV40Tag mice significantly inhibited the growth of autochthonous TSC tumors. **d** This inhibition of TSC tumor growth was accompanied by significantly lower testicular weights when the experiment was terminated at 77 weeks of age. Error bars indicate ± SE, and asterisks indicate significance

Male SJL.AMH-SV40Tag transgenic mice that develop autochthonous TSC tumors also express the MHC H-2^s^ haplotype that allows them to respond by proliferation to the immunodominant IA^s^-restricted Inα 215-234 peptide (Fig. 5a). The immunogenicity of Inα 215-234 in SJL.AMH-SV40Tag transgenic mice was confirmed by ELISPOT analysis of LNC taken 10 days after immunization with Inα 215-234. Whole LNC and CD4+ T cells but not CD8+ T cells purified from the primed LNC by magnetic bead separation showed high frequencies of antigen-specific IFNγ-secreting T cells in recall responses to Inα 215-234 (Fig. 5b). To determine whether vaccination against Inα 215-234 could inhibit the growth of autochthonous TSC tumors, male SJL.AMH-SV40Tag mice were vaccinated at eight weeks of age with Inα 215-234 in CFA or with CFA alone as control. We found that Inα 215-234 vaccination provided significant protection (*P* < 0.001) against the emergence and growth of autochthonous TSC tumors (Fig. 5c). This growth inhibition was confirmed when Inα 215-234 vaccinated mice showed significantly lower testicular weights (*P* = 0.02) when the experiment was terminated at 77 weeks of age (Fig. 5d).

## Discussion

We have shown that a targeted immune response against inhibin-α is capable of inhibiting the growth of TSC tumors in mice. Inhibition of tumor growth occurred using both therapeutic and prophylactic protocols and was effective against both autochthonous and transplantable tumors. Inhibin-α vaccination inhibited TSC tumor growth in both SJL/J and BALB/c mice and the effectiveness was associated with tumor infiltrates of activated CD4+ T cells. Moreover, the tumor immunity could be transferred into naive recipients with inhibin-α primed CD4+ T cells, B cells, or serum but not with primed CD8+ T cells.

Although there is a shortage of data showing the immunogenicity of inhibin-α in humans, several studies have shown substantial immunogenicity of inhibin-α following immunization of mice, rats, goats, sheep, and cattle [20–23]. These studies suggest that inhibin-α would be similarly immunogenic in humans and therefore likely useful as a vaccine target for immunotherapy of human TSC tumors.

It is noteworthy that transfer of either primed CD4+ T cells (Fig. 3a), B cells (Fig. 3e), or sera (Fig. 3g) on the day of tumor inoculation provides superior tumor immunity when compared to active immunization on the day when tumors become palpable (Fig. 2b). We believe that this is due primarily to the fact that in the transfer model, a mature effector immune response immediately engages the inoculated tumor cells whereas in the therapeutic active immunization model, immune engagement of the palpable tumor is delayed until a mature effector immune response develops several days if not weeks after vaccination. Thus the adoptive and passive transfer data mimic the tumor immunity provided when the active immunization occurs long before the inoculation of tumor (Fig. 2a).

It is also worth noting that the tumor immunity provided by transfer of primed CD4+ T cells is substantially enhanced when compared to the immunity provided by transfer of equal numbers of primed B cells or by transfer of primed sera. This enhanced tumor immunity suggests that helper function may not be the only way CD4+ T cells contribute to the TSC tumor immunity. CD4+ T cells can directly kill MHC class II positive tumor cells in a perforin/granzyme B cell-dependent manner and can indirectly eliminate tumor cells that lack expression of MHC class II by cytokine-mediated activation of macrophages [24, 25]. Experiments to determine how CD4+ T cells mediate inhibition of TSC tumor growth are ongoing as are experiments to determine whether antibodies mediate inhibition of TSC tumor growth by neutralization of inhibin-α or by complement-dependent cytotoxicity.

Our data clearly show that prophylactic inhibin-α vaccination provides results that are far superior to those obtained by therapeutic vaccination when the tumor has already grown and developed (Fig. 2a and b). Moreover, it is our view that virtually all vaccines function best when used prophylactically and that the earlier the immunity is provided, the better is the clinical outcome. This view is supported by the remarkable success of childhood vaccines that are designed to prevent rather than treat infectious diseases like polio, measles, and mumps. Though dramatically effective in preventing disease, these traditional childhood vaccines have little impact on viral clearance when vaccination occurs after infection and have no detectable efficacy in alleviating disease when vaccination occurs after the appearance of clinical signs [26].

The autoimmune consequences of inhibin-α vaccination must be considered. In normal human male tissues, high levels of inhibin-α expression is confined to the Sertoli cells and Leydig cells of the testis and to the glandular cells of the adrenal gland [27]. Inhibin-α negatively regulates pituitary secretion of follicle stimulating hormone (FSH) and has been shown to affect several other processes including cell proliferation, apoptosis, immunity, steroid hormone production, spermatogenesis, and germ cell development [4, 28]. Although inhibin-α vaccination may be useful in inhibiting growth and metastasis of human TSC tumors, the tradeoff for this tumor immunity may involve testicular and adrenal autoimmunity due to interference with a variety of cell processes affecting these tissues. In our prior studies we showed that inhibin-α vaccination of normal female mice resulted in antibody-mediated neutralization of inhibin-α that resulted in unregulated FSH release, enhanced litter numbers, early depletion of the ovarian reserve, and ultimately premature ovarian failure [14]. We have not similarly characterized the inhibin-α autoimmune phenotype in male mice. However, prior studies suggest that the autoimmune consequences of inhibin-α vaccination may be relatively benign and quite tolerable. No differences were detected in the interstitial tissue area between control and inhibin-α immunized animals [29]. However, inhibin-α immunization does affect peak testosterone levels but testosterone is still produced in quantities sufficient to facilitate secretion of gonadotropins [30]. Thus, the published evidence indicates that Leydig cells appear to be present and quite functional following inhibin-α immunization.

The current version of the inhibin-α vaccine is prototypic and could be improved substantially with a few simple refinements. Booster vaccinations may optimize the generated immunity and thereby provide greater inhibition of tumor growth. The unresolved granulomas associated with CFA preclude its use for human vaccination and will have to be replaced by a less toxic adjuvant that may still orchestrate a broad proinflammatory immune response involving Th1, Th17, and Th9 T cells implicated in the induction of aggressive tumor immunity [31].

It is somewhat surprising that the autochthonous TSC tumors that develop in male SJL.AMH-SV40Tag transgenic mice are of Leydig cell origin. In their original report, Dutertre and colleagues [11] showed that expression of the AMH-SV40Tag transgene in mice resulted in TSC tumors of Sertoli cell origin. However, after backcrossing for more than 20 generations from the original C57BL/6 background to the SJL/J background, the resultant autochthonous tumors clearly showed morphologic and histologic characteristics more consistent with Leydig cell tumors and expressed the luteinizing hormone receptor (LHR), a cell surface marker traditionally associated with Leydig cells rather than Sertoli cells [17]. Moreover, dysplasia in the SJL.AMH-SV40Tag transgenic mouse was consistently observed in cells within the interstitial space between seminiferous tubules where Leydig cells are located (Fig. 4b) and their derived tumors were identified by their characteristic polygonal shape with a large, granular cytoplasm and round nuclei with single or multiple nucleoli [32]. We did not observe dysplastic cells within the seminiferous tubules where Sertoli cell tumors typically develop as clusters of large tumor cells with eccentric nuclei and irregularly shaped cell membranes [33]. Thus, the well-documented, established characteristics of Leydig cell tumors correlated well with the observed features of the autochthonous TSC tumors that develop in SJL.AMH-SV40Tag transgenic mice.

All this being said, the expression of anti-Müllerian hormone (AMH) in Leydig cells remains a bit of an anomaly since AMH expression has traditionally been associated with Sertoli cell tumors rather than Leydig cell tumors [18] and AMH expression in normal cells is widely believed to be confined to Sertoli cells and not to Leydig cells [34–37]. However, the AMH-SV40Tag transgene is clearly expressed in both Leydig cells and Sertoli cells of SJL.AMH-SV40Tag transgenic mice (Fig. 4e) thereby implying that both cell types may express AMH and that transgene expression of SV40Tag may predispose to creation of tumors of either cell type with Sertoli cell tumors predominating in the original AT-t94 transgenic mouse generated in the C57BL/6 (H-2^b^) background strain and Leydig cell tumors predominating when the AMH-SV40Tag transgene is expressed in the SJL/J (H-2^s^) background strain. Indeed, strain-specific predispositions may occur as a result of differential epigenetic factors that are capable of shaping genetic outcomes [38].

TSC tumors of the testis comprise less than 5% of testicular cancers, and this rareness predisposes to being overlooked with respect to developing and testing new therapies for such uncommon tumors [3]. However, stage I TSC tumors have 5 year overall survivals that are significantly lower compared to those of stage I germ cell tumors that represent the vast majority of human testicular cancers [3]. Thus, though relatively rare, the enhanced lethality of TSC tumors begs for an improvement in treatment and clinical control. The results of our current study indicate that inhibin-α vaccination may be useful and effective as an adjuvant therapy for TSC tumors particularly since the vast majority of TSC tumors express inhibin-α [5–9] and that these tumors occur in young men in the prime of their life at a median age of 43 years [3].

## Conclusions

We have developed and characterized an immunotherapy for the most lethal forms of testicular cancer, namely TSC tumors that include Leydig cell tumors and Sertoli cell tumors. Inhibin-α vaccination provides an immunotherapeutic option that is currently unavailable for patients with these lethal forms of testicular cancer. If administered as adjuvant therapy along with current standard of care, inhibin-α vaccination may strengthen the natural immune defense against TSC tumors and thereby eliminate any residual local or systemic tumor cells. This novel way to control TSC tumors may have a substantial impact on the young men in the prime of their lives who are typically affected by this disease.

## Abbreviations

AMH: Anti-Müllerian hormone
AIRE: Autoimmune regulator transcription factor
CFA: Complete Freund's adjuvant
cpm: Counts per minute
Cy5: Cyanine 5
FITC: Fluorescein isothiocyanate
FSH: Follicle stimulating hormone
HPLC: High performance liquid chromatography
IFNγ: Interferon gamma
IPTG: Isopropylthiogalactopyronidase
LNC: Lymph node cells
LHR: Luteinizing hormone receptor
MHC: Major histocompatibility complex
Ni-NTA: Nickel-nitrilo triacetic acid
PE: Phycoerythrin
rmInα: Recombinant mouse inhibin-α
RT-PCR: Reverse transcription-polymerase chain reaction
SV40Tag: Simian virus 40 large transforming antigen
TSC: Testicular stromal cell
TILs: Tumor infiltrating lymphocytes

## References

[1] Howlader N, Noone AM, Krapcho M, Miller D, Bishop K, Altekruse SF, Kosary CL, Yu M, Ruhl J, Tatalovich Z, Mariotto A, Lewis DR, Chen HS, Feuer EJ, Cronin KA (eds). SEER Cancer Statistics Review, 1975-2013, National Cancer Institute. Bethesda, MD, http://seer.cancer.gov/csr/1975_2013/, based on November 2015 SEER data submission, posted to the SEER web site, April 2016.

[2] SEER Cancer Statistics Factsheets. Testis Cancer. National Cancer Institute. Bethesda, MD. http://seer.cancer.gov/statfacts/html/testis.html

[3] Banerji JS, Odem-Davis K, Wolff EM, Nichols CR, Porter CR (2016). Patterns of care and survival outcomes for malignant sex cord stromal testicular cancer: Results from the National Cancer Data Base. J Urol.

[4] Hedger MP, Winnall WR (2012). Regulation of activin and inhibin in the adult testis and the evidence for functional roles in spermatogenesis and immunoregulation. Mol Cell Endocrinol.

[5] Kommoss F, Oliva E, Bittinger F, Kirkpatrick CJ, Amin MB, Bhan AK, Young RH, Scully RE (2000). Inhibin-alpha CD99, HEA125, PLAP, and chromogranin immunoreactivity in testicular neoplasms and the androgen insensitivity syndrome. Hum Pathol.

[6] Taniyama H, Hirayama K, Nakada K, Numagami K, Yaosaka N, Kagawa Y, Izumisawa Y, Nakade T, Tanaka Y, Watanabe G, Taya K (2001). Immunohistochemical detection of inhibin-alpha, -betaB, and -betaA chains and 3beta-hydroxysteroid dehydrogenase in canine testicular tumors and normal testes. Vet Pathol.

[7] Yu C-H, Hwang D-N, Yhee J-Y, Kim J-H, Im K-S, Nho W-G, Lyoo Y-S, Sur J-H (2009). Comparative immunohistochemical characterization of canine seminomas and Sertoli cell tumors. 2009. J Vet Sci.

[8] Zheng W, Sung CJ, Hanna I, DePetris G, Lambert-Messerlian G, Steinhoff M, Lauchlan SC (1997). Alpha and beta subunits of inhibin/activin as sex cord-stromal differentiation markers. Int J Gynecol Pathol.

[9] Iczkowski KA, Bostwick DG, Roche PC, Cheville JC (1998). Inhibin A is a sensitive and specific marker for testicular sex cord-stromal tumors. Mod Pathol.

[10] Dudley A, McKinstry W, Thomas D, Best J, Jenkins A (2003). Removal of endotoxin by reverse phase HPLC abolishes anti-endothelial cell activity of bacterially expressed plasminogen kringle 5. Biotechniques.

[11] Dutertre M, Rey R, Porteu A, Josso N, Picard JY (1997). A mouse Sertoli cell line expressing anti-Müllerian hormone and its type II receptor. Mol Cell Endocrinol.

[12] Dutertre M, Gouédard L, Xavier F, Long WQ, di Clemente N, Picard JY, Rey R (2001). Ovarian granulosa cell tumors express a functional membrane receptor for anti-Müllerian hormone in transgenic mice. Endocrinology.

[13] Altuntas CZ, Jaini R, Kesaraju P, Jane-wit D, Johnson JM, Covey K, Flask CA, Dutertre M, Picard JY, Tuohy VK (2012). Autoimmune mediated regulation of ovarian tumor growth. Gynecol Oncol.

[14] Altuntas CZ, Johnson JM, Tuohy VK (2006). Autoimmune targeted disruption of the pituitary-ovarian axis causes premature ovarian failure. J Immunol.

[15] Yasamura Y, Tashjian AH, Sato GH (1966). Establishment of four functional, clonal strains of animal cells in culture. Science.

[16] Shin SI (1967). Studies on interstitial cells in tissue culture: steroid biosynthesis in monolayers of mouse testicular interstitial cells. Endocrinology.

[17] Shan LX, Hardy MP (1992). Developmental changes in levels of luteinizing hormone receptor and androgen receptor in Leydig cells. Endocrinology.

[18] Rey R, Sabourin JC, Venara M, Long WQ, Jaubert F, Zeller WP, Duvillard P, Chemes H, Bidart JM (2000). Anti-Müllerian hormone is a specific marker of Sertoli- and granulosa-cell origin in gonadal tumors. Hum Pathol.

[19] Ciaputa R, Nowak M, Madej JA, Poradowski D, Janus I, Dziegiel P, Gorzynska E, Kandefer-Gola M (2014). Inhibin-α, E-cadherin, calretinin and Ki-67 antigen in the immunohistochemical evaluation of canine and human testicular neoplasms. Folia Histochem Cytobiol.

[20] Forage RG, Brown RW, Oliver KJ, Atrache BT, Devine PL, Hudson GC, Goss NH, Bertram KC, Tolstoshev P, Robertson DM, de Kretser DM, Doughton B, Burger HG, Findlay JK (1987). Immunization against an inhibin subunit produced by recombinant DNA techniques results in increased ovulation rate in sheep. J Endocrinol.

[21] Morris DG, Browne D, Diskin MG, Sreenan JM (1997). Effect of peptide to carrier ratio on the immune and ovarian response to inhibin immunization in cattle. Anim Reprod Sci.

[22] Hennies M, Voglmayr JK, Dietrich E, Stollmann M, Moeller R, Holtz W (2001). Hormonal response of female goats to active immunization against a recombinant human inhibin alpha-subunit, and establishment of an enzyme-linked immunosorbent assay for caprine follicle-stimulating hormone. Reprod Domest Anim.

[23] Delves PJ, Lund T, Roitt IM (2002). Antifertility vaccines. Trends Immunol.

[24] Kennedy R, Celis E (2008). Multiple roles for CD4+ T cells in anti-tumor immune responses. Immunol Rev.

[25] Haabeth OA, Tveita AA, Fauskanger M, Schjesvold F, Lorvik KB, Hofgaard PO, Omholt H, Munthe LA, Dembic Z, Corthay A, Bogen B (2014). How Do CD4(+) T Cells Detect and Eliminate Tumor Cells That Either Lack or Express MHC Class II Molecules?. Front Immunol.

[26] Hildesheim A, Herrero R, Wacholder S, Rodriguez AC, Solomon D, Bratti MC, Schiller JT, Gonzalez P, Dubin G, Porras C, Jimenez SE, Lowy DR, Costa Rican HPV Vaccine Trial Group (2007). Effect of human papillomavirus 16/18 L1 viruslike particle vaccine among young women with preexisting infection: a randomized trial. JAMA.

[27] Uhlén M, Fagerberg L, Hallström BM, Lindskog C, Oksvold P, Mardinoglu A, Sivertsson Å, Kampf C, Sjöstedt E, Asplund A, Olsson I, Edlund K, Lundberg E, Navani S, Szigyarto CA, Odeberg J, Djureinovic D, Takanen JO, Hober S, Alm T, Edqvist PH, Berling H, Tegel H, Mulder J, Rockberg J, Nilsson P, Schwenk JM, Hamsten M, von Feilitzen K, Forsberg M, Persson L, Johansson F, Zwahlen M, von Heijne G, Nielsen J, Pontén F (2015). Proteomics. Tissue-based map of the human proteome. Science.

[28] Suresh PS, Rajan T, Tsutsumi R (2011). New targets for old hormones: inhibins clinical role revisited. Endocr J.

[29] Lovell TM, Knight PG, Groome NP, Gladwell RT (2000). Measurement of dimeric inhibins and effects of active immunization against inhibin α-subunit on plasma hormones and testis morphology in the developing cockerel. Biol Reprod.

[30] Voglmayr JK, Mizumachi M, Washington DW, Chen CL, Bardin CW (1990). Immunization of rams against human recombinant inhibin alpha-subunit delays, augments, and extends season-related increase in blood gonadotropin levels. Biol Reprod.

[31] Ivanova EA, Orekhov AN (2015). T helper lymphocyte subsets and plasticity in autoimmunity and cancer: an overview. Biomed Res Int.

[32] Al-Agha OM, Axiotis CA (2007). An in-depth look at Leydig cell tumor of the testis. Arch Pathol Lab Med.

[33] Chang B, Borer JG, Tan PE, Diamond DA (1998). Large-cell calcifying Sertoli cell tumor of the testis: case report and review of the literature. Urology.

[34] Guerrier D, Boussin L, Mader S, Josso N, Kahn A, Picard JY (1990). Expression of the gene for anti-Müllerian hormone. J Reprod Fertil.

[35] Rouiller-Fabre V, Carmona S, Merhi RA, Cate R, Habert R, Vigier B (1998). Effect of anti-Müllerian hormone on Sertoli and Leydig cell functions in fetal and immature rats. Endocrinology.

[36] Rajpert-De Meyts E, Jørgensen N, Graem N, Müller J, Cate RL, Skakkebaek NE (1999). Expression of anti-Müllerian hormone during normal and pathological gonadal development: association with differentiation of Sertoli and granulosa cells. J Clin Endocrinol Metab.

[37] La Marca A, Sighinolfi G, Radi D, Argento C, Baraldi E, Artenisio AC, Stabile G, Volpe A (2010). Anti-Mullerian hormone (AMH) as a predictive marker in assisted reproductive technology (ART). Hum Reprod Update.

[38] Koturbash I, Scherhag A, Sorrentino J, Sexton K, Bodnar W, Swenberg JA, Beland FA, Pardo-Manuel Devillena F, Rusyn I, Pogribny IP (2011). Epigenetic mechanisms of mouse interstrain variability in genotoxicity of the environmental toxicant 1,3-butadiene. Toxicol Sci.

